# Urinary stem cell-derived exocrine circRNA ATG7 regulates the SOCS1/STAT3 signaling pathway through miR-4500, inhibits M1 macrophage polarization, and alleviates the progression of diabetes nephropathy

**DOI:** 10.1007/s11255-023-03819-3

**Published:** 2023-10-10

**Authors:** Yang Sun, Yanhong Zhao, Yongxin Lu, Hongmei Li, Jin Xiang, Dongmei Yang, Jinrui Wang, Xinglian Gao, Yian Wang

**Affiliations:** Kidney Internal Medicine, The Sixth Affiliated Hospital of Kunming Medical University, Yuxi, 653100 Yunnan China

**Keywords:** Diabetes nephropathy, Urogenic stem cells, CircRNA ATG7, miR-4500, Macrophage

## Abstract

**Objective:**

The etiopathogenesis of diabetes nephropathy (DN) has not yet been fully clarified. Finding effective treatments to prevent renal failure in DN patients has become the main focus of research in recent years. Circular RNA (circRNA) has been shown to play a momentous role in DN progression. Based on this, we aimed to investigate the potential mechanism by which urine-derived stem cell (USC)-derived exosome circRNA ATG7 (Exo-ATG7) mediates DN progression.

**Methods:**

Exosomes from USCs were isolated and identified. The DN rat model was established by intraperitoneally injecting 60 mg/kg streptozotocin. The protein expression levels were measured by Western blot and immunofluorescence. HE and Masson staining were used to evaluate renal injury, and the expression of related genes was detected by RT-qPCR.

**Results:**

CircRNA ATG7 was significantly downregulated in the DN rat model, and the extracellular vesicles of USCs improved renal function and reduced inflammation in DN rats. However, after knocking down the USCs-derived exosome circRNA ATG7, improvement and therapeutic effect on renal function in DN rats were lost. In addition, overexpression of ATG7 facilitated the switching of macrophages from the pro-inflammatory M1 phenotype to the anti-inflammatory M2 phenotype both in vivo and in vitro. Mechanistically, upregulation of circRNA ATG7 expression can alleviate renal damage in DN rats. Importantly, the USCs-derived exosome circRNA ATG7 promotes macrophage M2 polarization by regulating the SOCS1/STAT3 signaling pathway through miR-4500. In addition, animal experiments also confirmed that after knocking down ATG7 in USC cells, the extracted exosome-treated DN rats could weaken the therapeutic effect of USC exosomes.

**Conclusion:**

Our research results indicate that USC-derived exosomal circRNA ATG7 facilitates macrophage phenotype switching from M1 to M2 through the SOCS1/STAT3 signaling pathway mediated by miR-4500, thereby inhibiting DN progression.

## Introduction

Diabetes (DM) is a modern common disease that threatens the lives’ of patients with this disease. It is caused by many factors and is accompanied by many complications [1]. Diabetes nephropathy (DN) is one of the most destructive DM microvascular complications and the main pathogenesis of end-stage renal disease (ESRD) [2]. DN pathogenesis is complex and mainly involves inflammation, oxidative stress, fibrosis, and the renin–angiotensin activation system (RAS) [3]. The main case characteristics of DN include glomerular basement membrane incrassation, mesangial matrix jacking, tubulo-interstitial fibrosis, and podocyte deficiency [4]. Although rigorous blood glucose, pressure control for DN patients, and the use of renin–angiotensin aldosterone system (RAAS) blockers are good strategies to slow down the development of disease, they cannot prevent or reverse the disease [5]. Therefore, the development of new remedy methods targeting the pathological mechanisms of DN has become the main focus.

Macrophages, as a module of innate immunity, play a momentous role in sustaining homeostasis in the body [6]. Under multifarious stimulation conditions, macrophages can differentiate into the “classically activated” pro-inflammatory M1 phenotype and the “alternatively activated” anti-inflammatory M2 phenotype [6, 7]. Recent research shows that Yi-Shen-Hua-Shi granules can inhibit DN by alleviating the damage to podocytes incurred by macrophage-derived exosomes [8]. Fenofibrate, as an agonist of PPAR-α, can restrain M1 macrophages by facilitating endothelial cell function in diabetic mice, thus preventing DN [9]. Therefore, we can prevent occurrence and development of DN by regulating macrophage polarization to interfere with the inflammatory reaction.

In recent years, stem cells have been widely used in the remedy of multifarious diseases and have been shown to efficaciously prevent DN progression [10]. Urinary-derived stem cells (USCs) are a subset of cells that are segregated from urine and have unique advantages due to their noninvasive acquisition method, strong proliferation capacity, and easy cultivation [11]. However, increasing evidence shows that the therapeutic effect of stem cells is exerted through their paracrine signaling mechanism, especially extracellular vesicles (EVs). Exosomes are the most hackneyed EVs, with a diameter of approximately 50–140 nm, and are capable of mediating intercellular communication by carrying RNA and proteins between cells or remote organs [12, 13]. Extracellular vesicles derived from USCs have been shown to prevent DN damage [14]. However, it remains unclear whether and how USCs-derived exosomes regulate macrophage polarization in the pathogenesis of DN.

Circular RNA (circRNA) is a covalent closed circular RNA that lacks 5′ and 3′ polarity compared to linear RNA [15]. Research has shown that circRNAs may regulate the involvement of microRNAs (miRNAs) in the progression of DN through sponge adsorption. The expression of circRNA_15698 was significantly upregulated in DN rats, and circRNA_15698 upregulated TGF-β1 expression by targeting miR-185 and promoted extracellular matrix (BCM) accumulation and fibrosis in DN [16]. CircRNA ATG7, also known as circ_0064288, is produced by the transcript of autophagy-associated gene 7 (ATG7), and studies have found that circRNA ATG7 can regulate the expression of ATG7 and participate in autophagy [17]. Many studies have shown that ATG7-mediated autophagy can regulate DN progression [18, 19]. However, expression pattern, biological function, and downstream competitive endogenous RNA (ceRNA) mechanism of circRNA ATG7 in DN remain unclear and largely unknown, and evidence for the involvement of exosome circRNA ATG7 in DN progression is limited.

In our study, we aimed to explore the role of the USCs-derived exosome circRNA ATG7 in DN. The USCs-derived exosome circRNA ATG7 may facilitate macrophage M2 polarization and affect DN progression. This pathway may become a new method to alleviate DN progression.

## Materials and methods

### USC isolation, culture, and identification

Using sterile containers, 150 mL of middle urine was collected from 6 healthy male donors (aged 26–28), and then 500 μL of antibiotic antifungal solution (Solarbio, Beijing, China) was added to a sterile container. All volunteers signed an informed consent form before conducting any research. The urine was centrifuged at 400×*g* for 10 min, the supernatant was discarded, and the cell precipitate was re-suspended in phosphate-buffered saline (PBS). The urine was centrifuged again at 200×*g* for 10 min and the supernatant was carefully removed. The bottom precipitated cells were re-suspended in the primary culture medium (Lonza, Walkersville, Maryland, USA) used for cultivating USCs and transferred to a 12-well plate. In subsequent experiments, only 2–6 generations of USCs were used.

Identify surface antigens of USCs by flow cytometry. USCs were immobilized for 12 h in ethanol (4 °C). Before assaying, USCs were flushed 3 times with PBS, and 50 μL of cell suspension was mixed with 50 μL of diluted positive surface antigen antibodies CD29-PE, CD90-APC and CD44-FITC and negative surface antigen antibodies CD45-FITC and CD34-APC and incubated at 4 °C for 25 min away from light. Then, the samples were centrifuged at 200×*g* at 4 °C for 5 min, the supernatant was discarded, and the washing process was repeated 3 times. The cells in 100 μL of PBS were detected by flow cytometry.

### Isolation and identification of exosomes (USC-Exos) from USC sources

The culture medium containing 80–90% fused USCs was removed, flushed with PBS 2–3 times, and then incubated in serum-free medium for another 48 h. The conditioned medium was collected and centrifuged at 400×*g* at 4 °C for 15 min. Then, the supernatant was centrifuged again at 2000×*g* for 20 min. Subsequently, a 0.22 µm filter was used to filter the supernatant to remove dead cells and cell debris. The samples were centrifuged at 100,000×*g* for another 120 min to deposit USC-exos. Ultimately, the precipitated USC-Exo particles were re-suspended at 200 μL in PBS. USC-Exos were evaluated by transmission electron microscopy (TEM). The exosome particles were immobilized in 3% (w/v) glutaraldehyde and 2% paraformaldehyde cacodylic acid salt buffer solution and loaded onto a copper grid coated with Formvar. After flushing and drying, the mesh was compared in 2% uranyl acetate and then checked by TEM. Exosome markers Alix, CD63, and Tsg101 were tested using Western blotting.

### Cell transfection

Cells were inoculated into a 6-well plate at 3 × 10^5^ cells/well. According to the specifications, si-ATG7, oe-ATG7, miR-4500 mimic, miR-4500 inhibitor, oe-ATG7 + miR-4500 mimic, and si-ATG7 + miR-4500 inhibitor were transfected into cells by Lipofectamine 2000 (Invitrogen, Carlsbad, California, USA). After 6 h, the transfected complex was removed and replaced with fresh culture substrate. After 48 h, the cells were collected for subsequent experiments.

### Cell culture

The mouse mononuclear macrophage line RAW264.7 was purchased from the China Typical Culture Collection Center. RAW264.7 cells were cultured in DMEM (Sigma‒Aldrich, St. Louis, Missouri, USA) substrate containing 10% FBS. Then, 100 ng/mL lipopolysaccharide (LPS) (Sigma‒Aldrich, St. Louis, Missouri, USA) for one day to induce the polarization of RAW264.7 cells.

### Detection of the double luciferase reporter gene

Predicted 3′-UTR sequences and mutation sequences within predicted targets for the interaction between circRNA ATG7 and miR-4500 or miR-4500 and SOCS1 were synthesized and inserted into pmirGLO control vectors (E1330, Promega, Madison, USA). A total of 50 nmol/L negative control, miR-4500 mimic, or 200 nmol/L miR-4500 inhibitor and 200 ng/µL wild-type or mutant 3′-UTR plasmid were co-transfected into HEK293T cells by Lipofectamine 2000. After transfection for 48 h, luciferase activity was measured by a Dual Luciferase Assay Kit (E1910, Promega, Madison, USA). Firefly luciferase activity was normalized to Renilla luciferase activity.

### Animal experiments

Male Sprague Dawley (SD) rats aged 6–8 weeks were purchased from the Animal Experiment Center of Kunming Medical University, and after one week of accommodative feeding, DM was induced by a single intraperitoneal injection of 60 mg/kg streptozotocin (STZ, S0130, Sigma, St. Louis, Missouri, USA) for five consecutive days. After 24 h of follow-up blood glucose examination, DM was confirmed with a blood glucose level of ≥ 16.7 mmol/L for 3 consecutive days. After 4–6 weeks of STZ treatment, urine was collected from the DM rats in metabolic cages, and volume and protein thickness were measured. Urinary protein ≥ 30 mg/24 h was deemed to indicate DN. The rats were randomly divided into the following groups: normal control group (0.1 M PBS solution was injected intraperitoneally into rats, the volume was the same as that of the model group, *n* = 15), DN group (the rats were injected with 60 mg/kg STZ intraperitoneally, *n* = 15), Exo group (USC cell-derived exosomes were injected into DN rats through the tail vein, *n* = 15), and sh-ATG7-Exo group (the DN rats were injected with exosomes that knocked down ATG7 through the tail vein, *n* = 15). After 4 weeks of treatment, urine specimens were collected from DN rats, and the rats were killed after being anesthetized with isoflurane. Then, rat kidney tissue was collected, and blood and urine were collected for renal function testing. Serum creatinine and serum blood urea nitrogen were measured with a kit (Jiancheng Bioengineering Institute, Nanjing, China) according to the specifications. Protein thickness in urine was measured by a BCA kit (Beyotime, Shanghai, China). All animal experiments were authorized by the Animal Ethics Committee of Kunming Medical University.

### Histological analysis

Kidney tissue from experimental rats was obtained, fixed with 4% paraformaldehyde for 24 h, dehydrated in different levels of alcohol, and cleared with xylene. The kidney tissue was embedded in paraffin wax and cut into 4 μm slices with a microtome. The sections were routinely dewaxed and hydrated prior to tissue staining. Then, HE, PAS, and Masson staining kits (Solarbio, Beijing, China) were used to stain the tissues separately according to the manufacturer's instructions. The stained slices were sealed with neutral gum. Finally, they were observed and photographed under a light microscope.

### Immunofluorescence staining

After dewaxing and rehydrating the sections of rat kidney tissue, the sections were rinsed with PBS and then treated in sodium citrate antigen repair solution (Solarbio, Beijing, China) at 95 °C for 30 min. After washing with PBS, the cells were treated with 3% hydrogen peroxide for 10 min to block endogenous enzymes. Then, 5% BSA was added to the slices and incubated at 25 °C for 2 h. For cells, the cells were fixed with 4% paraformaldehyde for 30 min, permeated with 0.5% Triton X-100 for 20 min, and then blocked with 5% BSA at room temperature for 1 h. Diluted F4/80 (1:100, Abcam, UK) and iNOS (1:100, Abcam, UK) antibodies were added to the samples and incubated in a wet box at 4 °C overnight. On the next day, the membrane was rewarmed at room temperature for 30 min and then incubated with fluorescein-labeled secondary antibody (1:1000, Abcam, UK) for 2 h. The cells were washed with PBS 3 times for 5 min each time, and the excess water was removed with absorbent paper. Then, DAPI (Invitrogen, CA, USA) was added and incubated for 10 min without light. After the anti-quenching sealing tablets were sealed, they were observed under a fluorescence microscope.

### RT-qPCR analysis

Total RNA was extracted from cells and tissues with an RNAsimple total RNA extraction kit (TIANGEN, Beijing, China), and then the RNA concentration and mass were measured. RNA specimens were treated with DNase I, and RNA was reverse-transcribed into cDNA. RT-PCR was performed with GAPDH and U6 as internal controls on the Bio-Rad CFX Connect platform. The detailed primer sequences are shown in Table 1, and differential gene expression was analyzed by the 2^−ΔΔ*C*t^ method.

**Table 1:** Primer sequences

Target	Sequence (F: forward prime, R: reversed prime)
CircRNA ATG7	F: 5′-CTCCCTCTTGACATTGCAGAGTG-3′
	R: 5′-GAGTCTTGAAAGACTCGAGTGTGTGG-3′
MiR-4500	F: 5′-GGGTGAGGGTAG-3′
	R: 5′-CAGTGCGTGTGTGGAGT-3′
GAPDH	F: 5′-GCAACTAGGATGGTGGTGGCT-3′
	R: 5′-TCCCATCCCACGCTCTCATA-3′
U6	F: 5′-CTCGCTTCGGCAGCACA-3′
	R: 5′-AACGTTCACGAATTTTGCGT-3′

### Western blotting

Total proteins were extracted by RIPA lysis buffer replenished with a protease inhibitor mixture (Beyotime, Shanghai, China), measured by BCA (Beyotime, Shanghai, China), and separated by Tris–glycine/SDS gel. The isolated proteins were transferred to PVDF membranes, and the PVDF membranes were blocked with 5% skim milk powder. Then, diluted primary antibody was added to the PVDF membranes. The antibodies were purchased from Abcam (UK). TNF-α (1:1000), IL-1β (1:1000), iNOS (1:1000), IL-10 (1:1000), Arg-1 (1:1000), CD63 (1:1500), Alix (1:1500), Tsg101 (1:1000), SOCS1 (1:500), p-STAT3 (1:1000), STAT3 (1:1500), and β-actin (1:10,000) were incubated overnight at 4 °C on a shaking bed. On the next day, after washing with TBST 3 times, HRP-labeled secondary antibody was added and incubated at room temperature for 1 h. ECL chemiluminescence was used for development, a chemiluminescence instrument was used for exposure and observation, and ImageJ was used for protein band analysis.

### Oil Red O staining to detect lipid content

Neutral lipids in USCs were obtained using an Oil Red O staining kit (Beyotime, Shanghai, China). The cells were flushed 3 times with PBS and immobilized for 15–30 min with 4% paraformaldehyde at 4 °C. After removing the fixative, the specimen was rinsed with PBS and dyed with oil red O. After 30–60 min, oil red O was removed, and the sample was washed with washing solution and double distilled water. Finally, the image was viewed using a microscope.

### Alizarin Red S staining

Cells were collected and cultured for 21 days. After three PBS washes, the USCs were fixed in 4% PFA (Beyotime, Shanghai, China) for 15 min. Then, the USCs were gently rinsed with distilled water to remove residual fixative and dyed with 1% Alizarin Red S (Sigma, St. Louis, Missouri, USA) for 10 min. Finally, pictures were taken with a light microscope.

### Statistical analysis

All data were analyzed using GraphPad Prism 8.0, and the analysis results are expressed as the mean ± SD. All experiments were executed in 3 parallel groups and repeated 3 times. In one-way ANOVA, T test is the means of analysis. *p* < 0.05 was considered statistically significant.

## Results

### Isolation and identification of USCs and characterization of USC-Exos

**Fig. 1:**
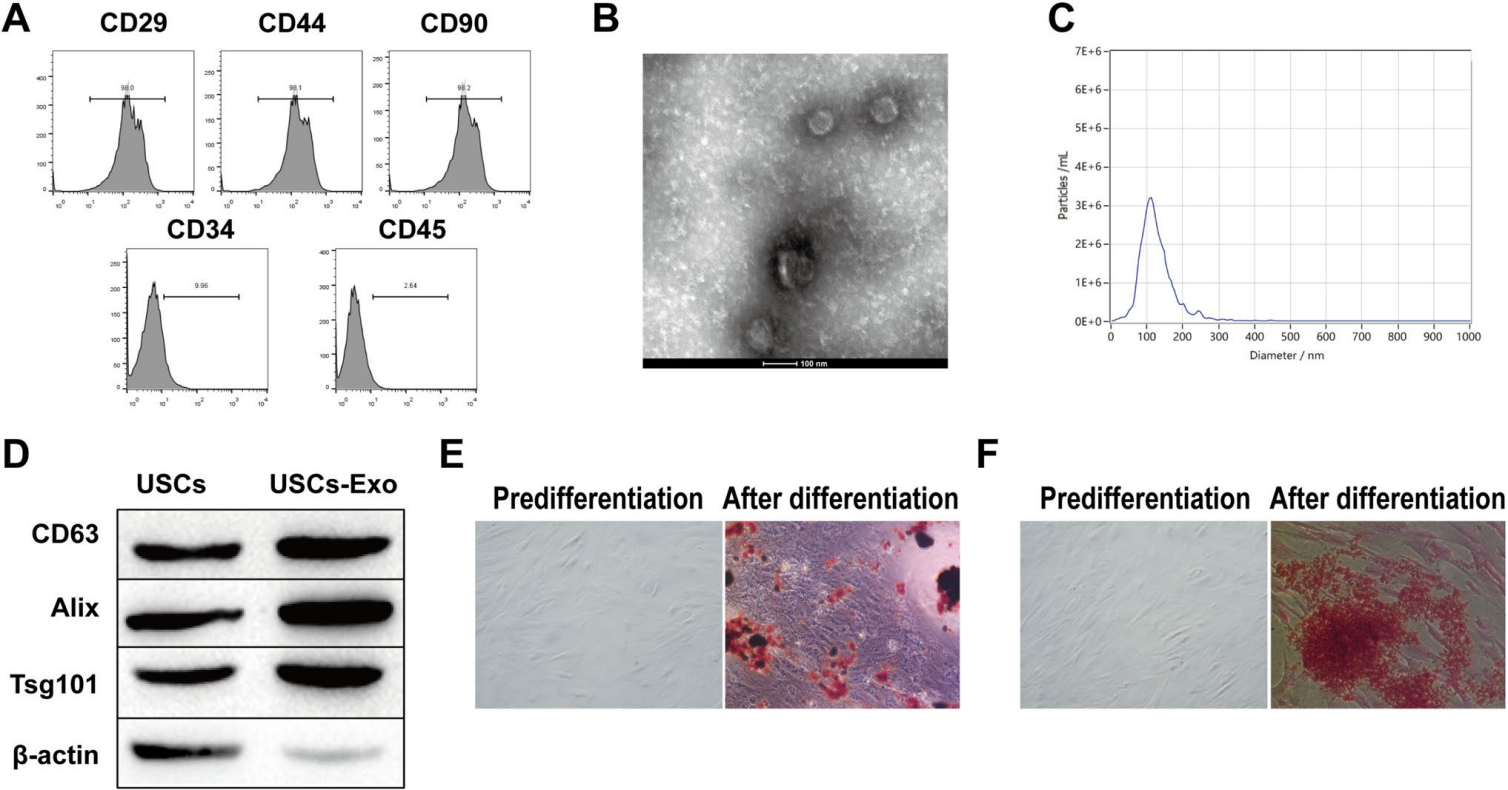
Isolation and Identification of USCs and Characterization of USC-Exos. **A** USCs surface markers were analyzed by flow cytometry; **B** Morphology of USCs-Exo was observed using TEM; **C** Nano Sight analysis measures the particle size of USCs-Exo; **D** Exosome markers were detected by Western blot. **E**–**F** Osteogenic and lipogenic differentiation ability of USCs was observed by alizarin red S and oil red O staining

USCs were segregated from 6 healthy male donors and analyzed for surface markers using flow cytometry. The results showed that CD44, CD90, and CD29 of USCs were positive, while CD34 and CD45 were negative (Fig. 1A). This indicates that we successfully isolated USCs. Subsequently, we extracted and identified USC-Exos. The morphology of USC-Exos was observed by TEM, and their size was measured by Nano Sight analysis. The results showed that USC-Exos are global vesicles (Fig. 1B). Particle size analysis showed that the size of USC-Exos was approximately 50–100 nm (Fig. 1C). Western blot analysis of exosome markers showed that CD63, Alix, and Tsg101 were highly expressed in USC-Exos (Fig. 1D), indicating that USC-Exos had been successfully isolated. In addition, the staining results of Alizarin Red S and Oil Red O showed that USCs showed high osteogenic and lipogenic differentiation ability (Fig. 1E and F).

### ATG7 was downregulated in the renal tissue of DN rats

**Fig. 2:**
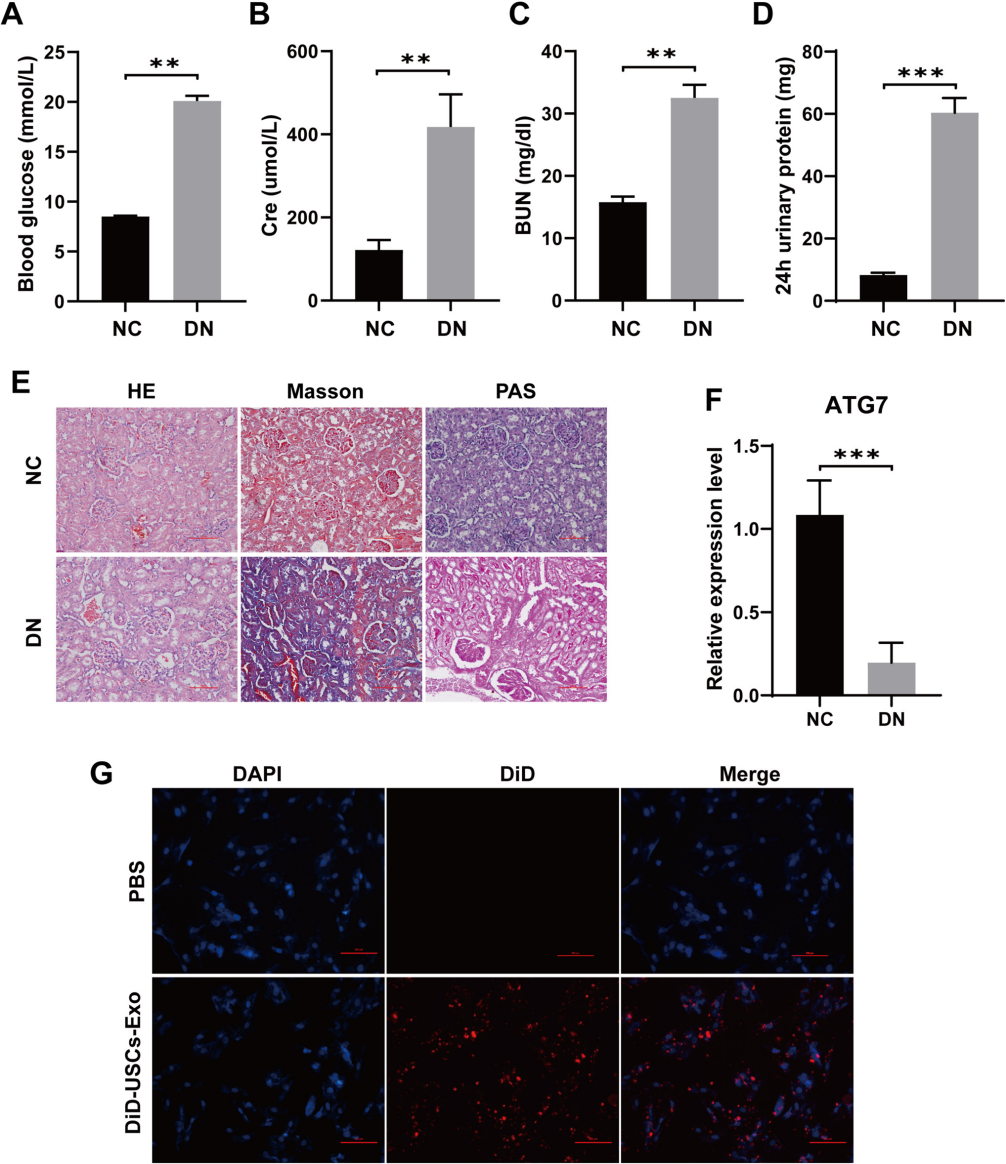
ATG7 was downregulated in the renal tissue of DN rats. **A** Blood glucose detection in rats; **B** Rat serum Cre detection; **C** Serum BUN detection in rats; **D** 24-h urine protein detection in rats; **E** The pathological changes in renal tissue were observed by HE, Masson and PAS staining; **F** The expression of ATG7 in renal tissue was detected by RT-qPCR; **G** Localization of DiD-labeled USC-Exos in renal tissue

We constituted a DN rat model by injecting STZ intraperitoneally. The results manifested that compared to the NC group, blood sugar, serum creatinine (Cre), serum blood urea nitrogen (BUN), and 24-h total urine protein of DN rats increased significantly (Fig. 2A–D). HE, PAS, and Masson staining results showed that compared to the NC group, DN rats exhibited increased renal tubular dilation, amassing of inflammatory cells in the interstitial region, glomerular dilatancy and sclerosis, and renal interstitial fibrosis in DN kidneys (Fig. 2E). These results indicate that the DN rat model was successfully established. In addition, we also used RT-qPCR to test ATG7 levels in renal tissue, and the expression of ATG7 was observably downregulated in DN rats (Fig. 2F). The localization of DiD-labeled USC-Exos in renal tissue showed that in the DN rat model, DiD-labeled USC-Exos were mainly enriched in renal tissue (Fig. 2G). The above data indicate that USC-Exos can be transferred to renal tissue and act as important cell regulators.

### USC-Exos can moderate renal damage in DN rats, while knocking down ATG7 facilitates renal damage in DN rats

**Fig. 3:**
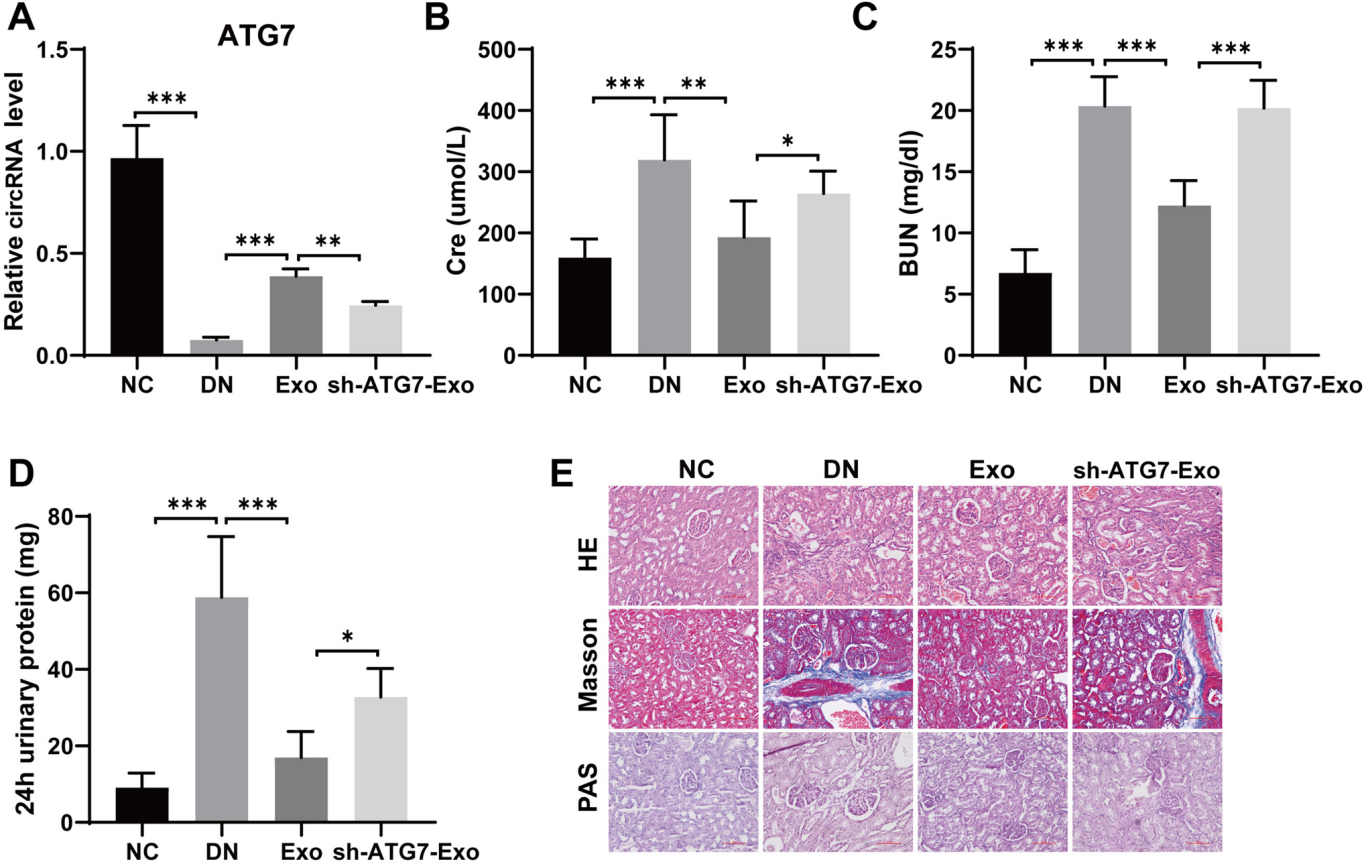
Knocking down ATG7 in USC-Exos can facilitate renal injury in DN rats. **A** RT-qPCR for checking ATG7 levels in renal tissue; **B** Rat serum creatinine detection; **C** Serum BUN detection in rats; **D** 24-h urine protein detection in rats; **E** HE, Masson, and PAS staining for observing renal tissue

Next, to verify the role of circRNA ATG7 knockdown USC-Exos (sh-ATG7-Exos) in DN rats, we first knocked down ATG7 in USC cells and then extracted USC exosomes that knocked down ATG7 to treat DN rats. RT-qPCR detection of ATG7 expression manifested that compared to that in the DN group, the level of ATG7 in the normal USC exosome treatment group increased, while ATG7 expression was significantly downregulated in the exosome group with ATG7 knockdown (Fig. 3A). The results of renal function-related test indicators showed that compared to the DN group, serum Cre, serum BUN, and 24 h total urinary protein of rats were observably lessened in the normal USC exosome treatment group. After treatment with exosomes that knocked down ATG7, the levels of these indicators increased (Fig. 3B–D). HE, PAS, and Masson staining manifested that compared to the normal USC exosome treatment group, the exosome group with ATG7 knockdown significantly increased renal tubular dilation, amassing of inflammatory cells in the interstitial region, glomerular dilatancy and sclerosis, and renal interstitial fibrosis in DN kidneys (Fig. 3E). In summary, our data suggest that USC-Exos can alleviate renal injury in DN rats, while knocking down ATG7 weakens the remedial influence of USC-Exos on renal damage in DN rats.

### Overexpression of ATG7 facilitates macrophage switch from M1 to M2 in vivo

**Fig. 4:**
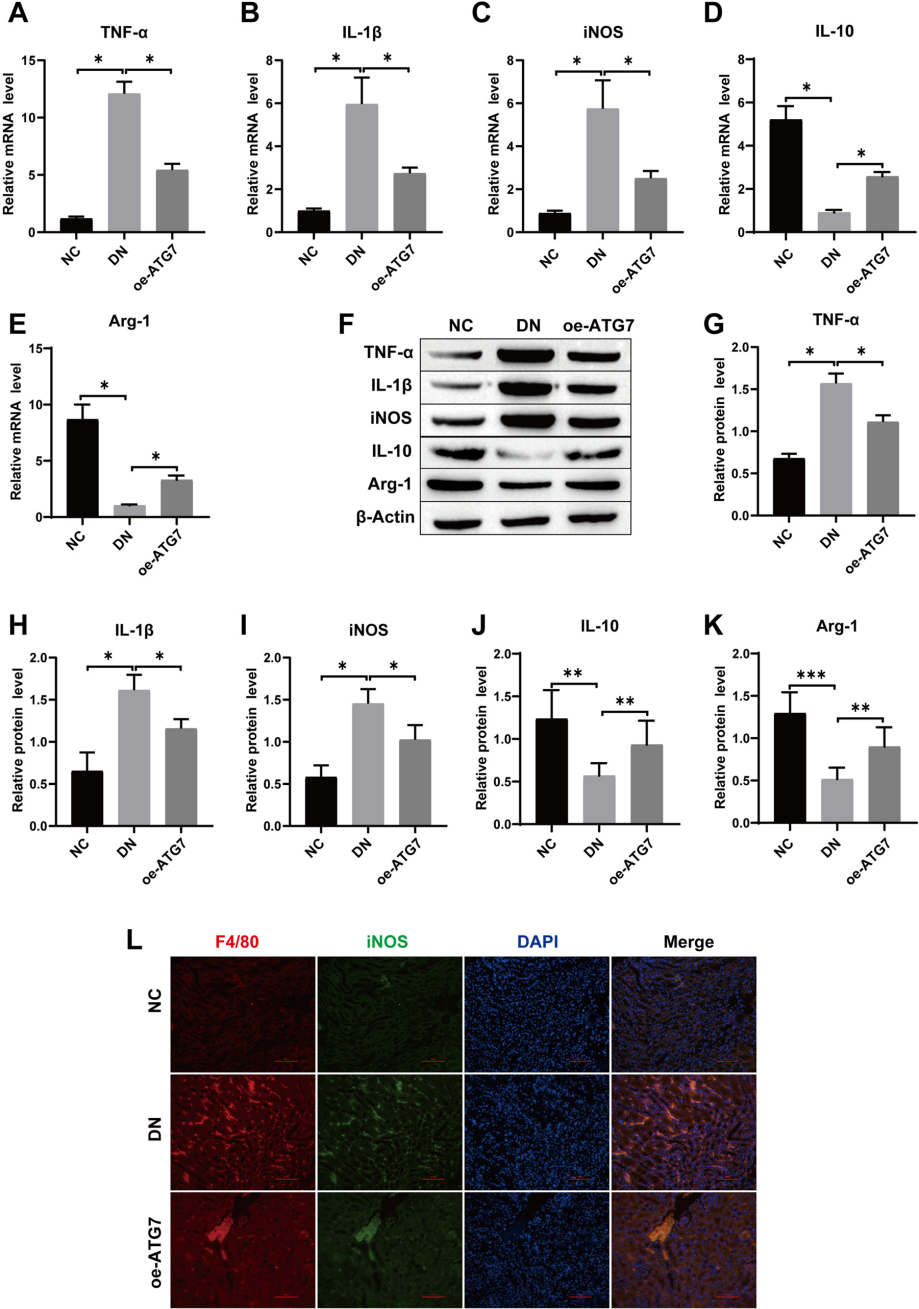
Overexpression of ATG7 promotes macrophage switching from M1 to M2 in vivo. **A**–**E** The mRNA expression levels of TNF-α, IL-1β, IL-10, iNOS and Arg-1 were detected by RT-qPCR; **F**–**K** The protein expression levels of TNF-α, IL-1β, IL-10, iNOS and Arg-1 were detected by Western blot; **L** F4/80 and iNOS double staining for detecting M1 phenotype polarization of macrophages in rat kidney tissue

It has been verified that DN pathogenesis is interrelated with persistent inflammation triggered by M1 macrophage revitalization. We treated DN rats with ATG overexpression and then observed the effect of ATG7 on macrophage polarization. RT-qPCR and Western blotting were used to detect the expression of the inflammatory factors TNF-α, IL-1β, and IL-10 and macrophage M1 markers (iNOS) and M2 markers (Arg-1). The results showed that compared with those in the NC group, the levels of TNF-α, IL-1β and iNOS in the DN group were increased, and the levels of the anti-inflammatory factors IL-10 and Arg-1 were decreased, while the malignant manifestations in the DN group were alleviated after overexpression of ATG7. (Fig. 4A–K). In addition, the expression level of iNOS, a marker of M1 macrophages, in renal tissues was detected by immunofluorescence staining. The results showed that the expression of iNOS was decreased after overexpression of ATG7, indicating that overexpression of ATG7 inhibited the polarization of M1 macrophages. (Fig. 4L).

### Overexpression of ATG7 facilitates macrophage switching from M1 to M2 in vitro

**Fig. 5:**
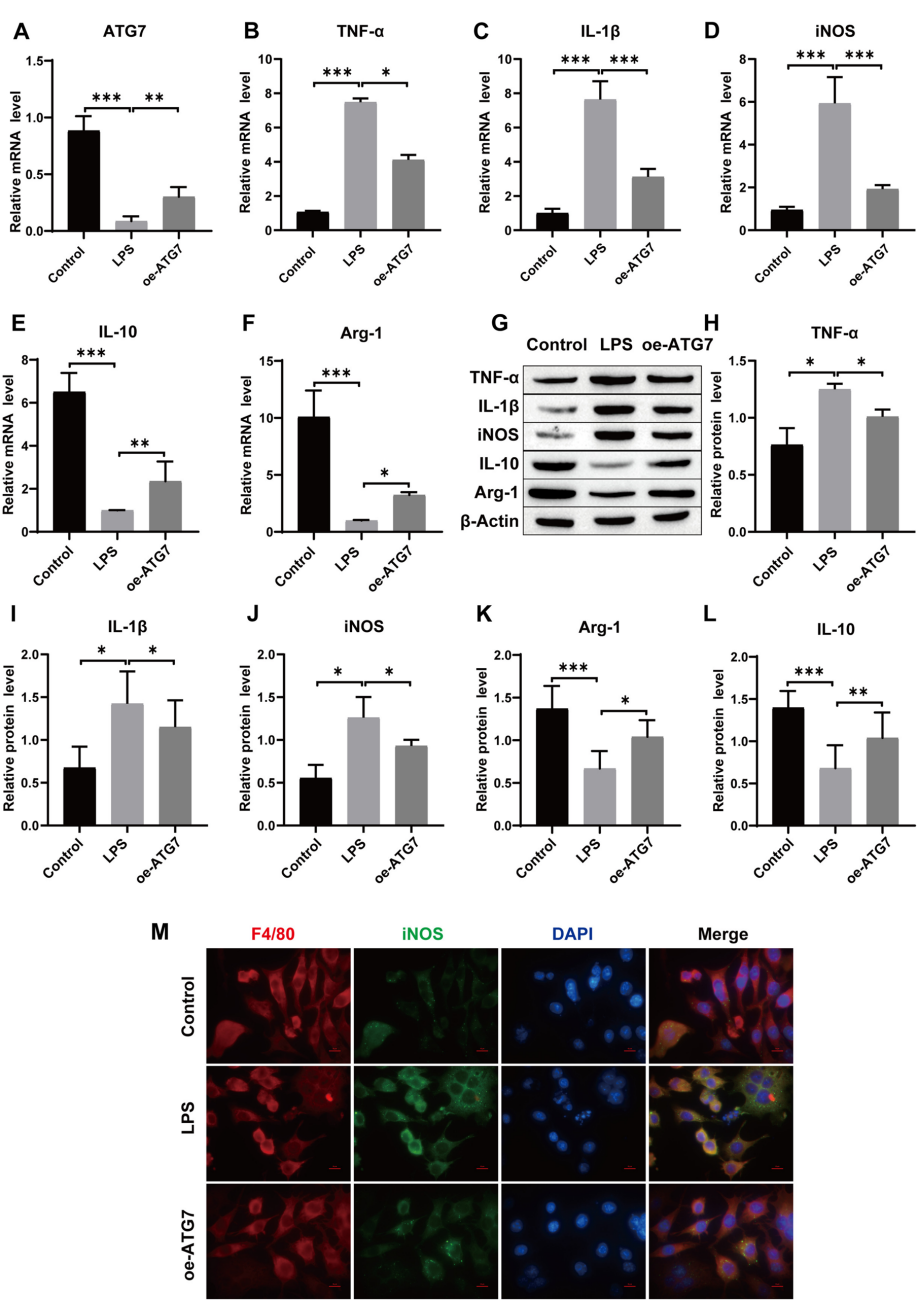
The effect of ATG7 on macrophage polarization in vitro. **A** RT-qPCR for determining ATG7 levels; **B**–**F**: The mRNA expression levels of TNF-α, IL-1β, IL-10, iNOS and Arg-1 were detected by RT-qPCR; **G**–**L** The protein expression levels of TNF-α, IL-1β, IL-10, iNOS and Arg-1 were detected by Western blotting; **M** F4/80 and iNOS double staining were used to detect M1 phenotype polarization in macrophages

Next further validation experiments were conducted in vitro. LPS was used to treat the macrophage line RAW264.7 to induce cell polarization. RT-qPCR showed that compared with that in the control group, LPS-induced ATG7 expression was significantly reduced, while overexpression of ATG7 upregulated ATG7 expression (Fig. 5A). Subsequently, RT-qPCR and Western blotting were used to measure the levels of inflammatory factors and the polarization of macrophages. The results showed that the expression levels of TNF-α, IL-1β and iNOS were significantly increased after LPS induction, and the mRNA and protein expression levels of IL-10 and Arg-1 were downregulated, while the effect of LPS was weakened by oe-ATG7 treatment (Fig. 5B–L). In addition, immunofluorescence staining showed similar results, with oe-ATG7 treatment reducing the LPS-induced increase in iNOS expression (Fig. 5M). In summary, our results showed that oe-ATG7 prominently restrains macrophage switching toward the M1 phenotype and facilitates macrophage switching toward the M2 phenotype.

### miR-4500 is a direct target of ATG7

**Fig. 6:**
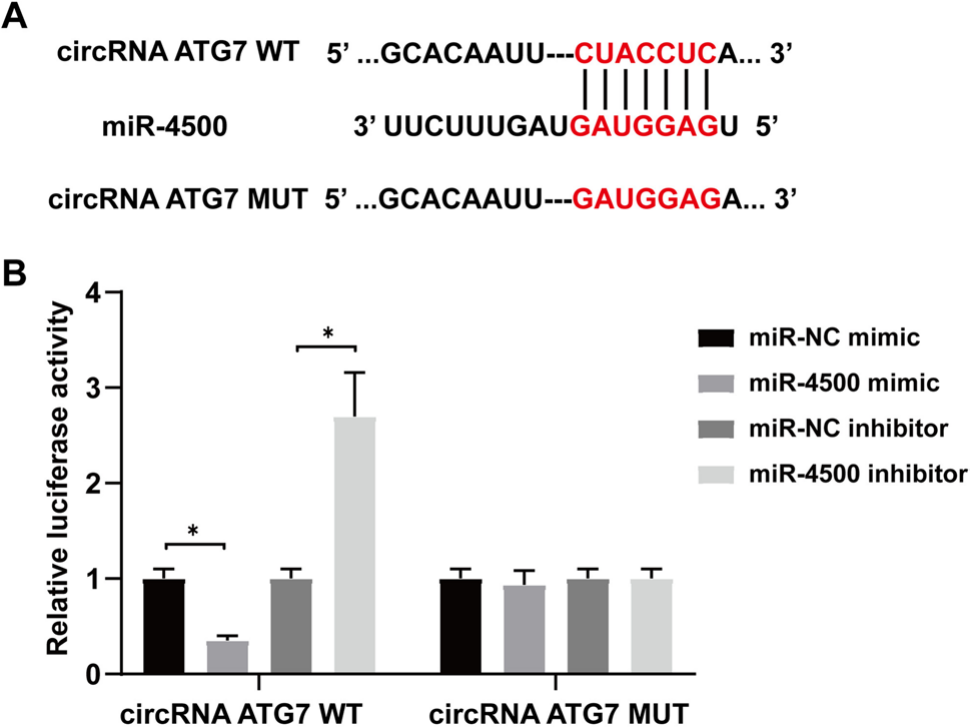
ATG7 directly targets miR-4500. **A** StarBase website was used to predict targeted combining sites; **B** Dual luciferase report assay certified the targeting relationship

StarBase (http://starbase.sysu.edu.cn) was used to predict that ATG7 has a specific binding site for miR-4500 (Fig. 6A). The double luciferase reporter gene assay showed that in the WT-ATG7 reporter gene, the miR-4500 mimic decreased luciferase activity, but the miR-4500 inhibitor increased luciferase activity. However, the MUT-ATG7 reporter gene showed no prominent change in luciferase activity (Fig. 6B). Therefore, miR-4500 is targeted by ATG7.

### miR-4500 regulates the SOCS1/STAT3 signaling pathway

**Fig. 7:**
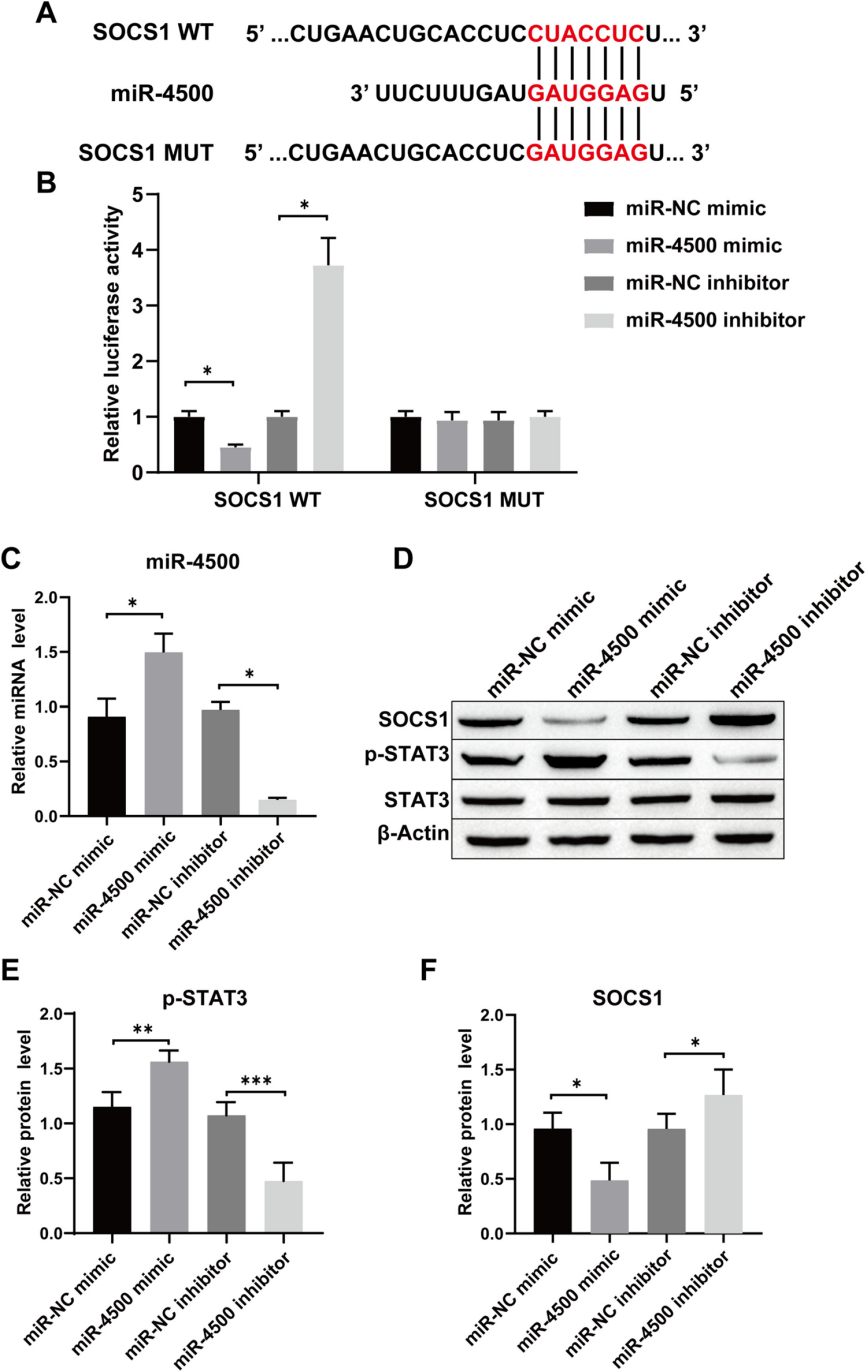
miR-4500 regulates the SOCS1/STAT3 signaling pathway. **A** StarBase website was used to predict targeted binding sites; **B** Double luciferase report assay to certify the targeting relationship; **C** The transfection efficiency of miR-4500 was verified by RT-qPCR; **D**–**F** Expression levels of SOCS1, p-STAT3 and STAT3 were detected by Western blot

StarBase (http://starbase.sysu.edu.cn) was used to predict that miR-4500 has a specific binding site for SOCS1 (Fig. 7A). The double luciferase reporter gene assay was performed with the WT-SOCS1 reporter gene. The miR-4500 mimic reduced the fluorescence activity, but the miR-4500 inhibitor increased the fluorescence activity. However, the MUT SOCS1 reporter gene showed no significant change in fluorescence activity (Fig. 7B). Therefore, miR-4500 can target SOCS1 binding. The expression results of miR-4500 showed that compared to the NC group, transfection with the miR-4500 mimic elevated the miR-4500 level in cells, while the miR-4500 inhibitor decreased the level of miR-4500 (Fig. 7C). Western blot analysis showed that the expression of SOCS1 protein in the miR-4500 mimic group was decreased and the expression of p-STAT3 was increased compared with the miR-NC mimic group. However, after transfection with the miR-4500 inhibitor, SOCS1 expression was increased, and p-STAT3 expression was decreased (Fig. 7D–F). In short, our results indicate that miR-4500 can target and regulate the SOCS1/STAT3 signaling pathway.

### Overexpression of ATG7 promotes the polarization of macrophages from M1 to M2 through regulation of the SOCS1/STAT3 signaling pathway by miR-4500

**Fig. 8:**
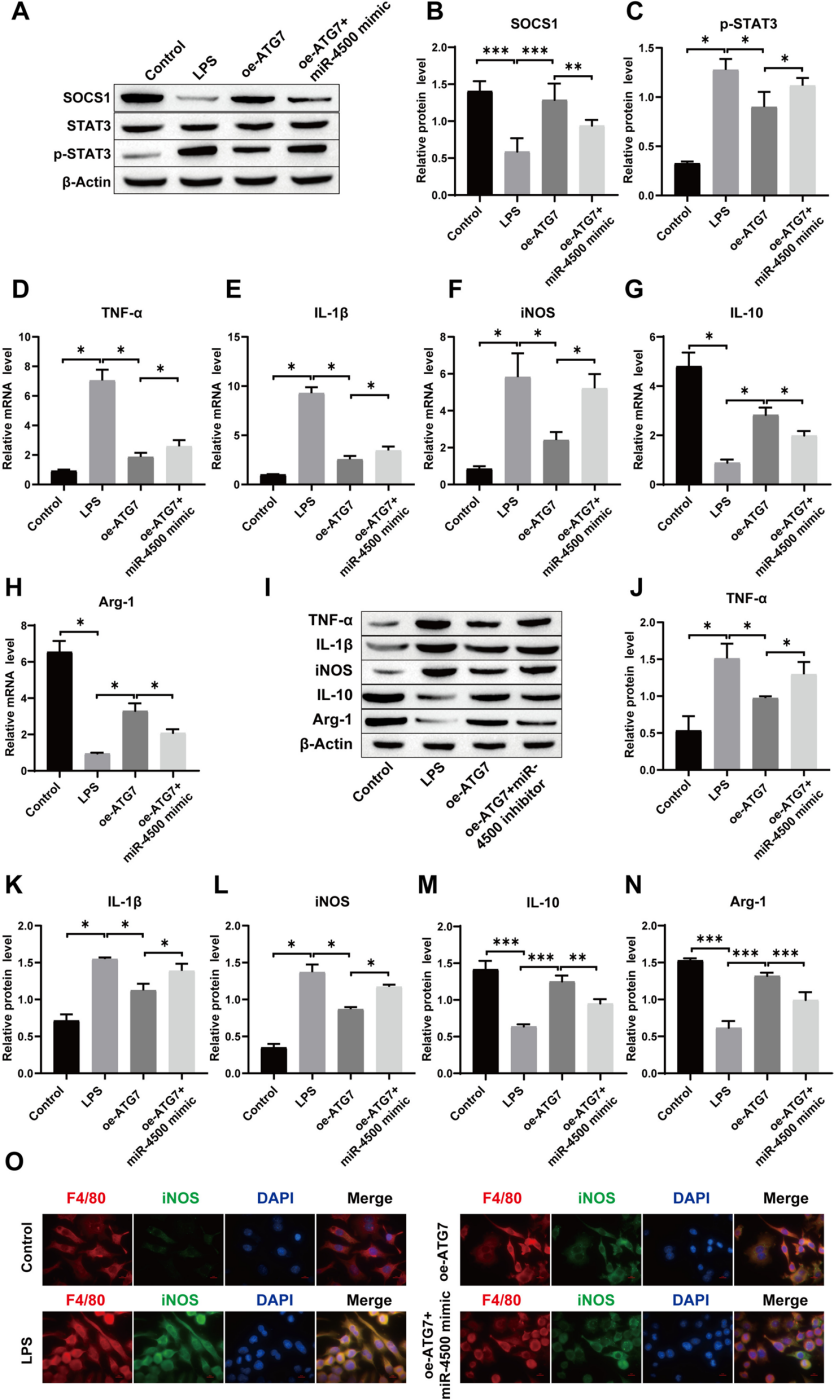
Overexpression of ATG7 affects macrophage polarization through regulation of the SOCS1/STAT3 signaling pathway by miR-4500. **A**–**C** Western blot for detecting SOCS1, p-STAT3 and STAT3 protein levels; **D**–**H** The mRNA expression levels of TNF-α, IL-1β, IL-10, iNOS and Arg-1 were detected by RT-qPCR; **I**–**N** The protein expression levels of TNF-α, IL-1β, IL-10, iNOS, and Arg-1 were detected by Western blot; **O** F4/80 and iNOS double staining for checking M1 phenotype polarization in macrophages

Next, we transfected oe-ATG7 and miR-4500 mimic into LPS-induced RAW264.7 cells to verify that ATG7 promotes macrophage polarization through the regulation of the SOCS1/STAT3 signaling pathway by miR-4500. First, Western blot results showed that compared to the LPS group, the expression of p-STAT3 protein was significantly decreased and the expression of SOCS1 protein was increased after overexpression of ATG7, while transfection of miR-4500 mimic significantly reversed this process (Fig. 8A–C). Subsequently, Western blotting and RT-qPCR were used to determine the changes in inflammatory factors and phenotypic markers of M1 and M2 macrophages. The results showed that compared to the LPS group, overexpression of ATG7 inhibited the expression of TNF-α, IL-1β and iNOS and promoted the expression of IL-10 and Arg-1. After transfection with the miR-4500 mimic, the effect of overexpressed ATG7 was significantly weakened. (Fig. 8D–N). In addition, immunofluorescence detection results showed that the miR-4500 mimic also reversed the reduced effect of oe-ATG7 on iNOS expression (Fig. 8O). In short, our results show that oe-ATG7 promotes the polarization of macrophages from M1 to M2 by regulating the SOCS1/STAT3 signaling pathway through miR-4500.

## Discussion

Diabetes nephropathy (DN) is the central cause of chronic kidney disease. DN pathogenesis is poly-factorial, involving multiple molecular pathways [20]. Persistent inflammation is one of the primary factors contributing to DN progression [21], and macrophages are key inflammatory cells mediating renal inflammation in both experimental and human DM. Therefore, inhibiting the polarization of macrophages toward the M1 pro-inflammatory phenotype is crucial for preventing the inflammatory microenvironment in DN. In this study, we successfully obtained USC-Exos, and their role in DN rats was studied. In vivo studies have shown that overexpression of circRNA ATG7 can significantly alleviate renal damage in DN rats and facilitate macrophage polarization from M1 to M2. Further experiments have shown that overexpression of circRNA ATG7 may alleviate renal injury by regulating the SOCS1/STAT3 pathway through miR-4500 and promoting macrophage switching from M1 to M2.

An increasing number of studies have shown that stem cell-derived exosomes can act not only as biomarkers for early DM diagnosis but also as latent remedial tools for DM and its complications [22, 23]. For instance, Dalirfardouei et al. [24] showed that exosomes (MSC-Exos) derived from mesenchymal stem cells promote the switching of macrophages to the M2 phenotype, thereby reducing the inflammation of diabetic foot ulcer wounds and promoting healing. In this paper, urinary stem cells are used as a treatment method to explore the progression of DN. Compared to other stem cells, modified stem cells have the superiorities of noninvasive material extraction, simple extraction and separation steps, higher telomerase activity and longer telomere sequences, good multidirectional differentiation potential, high safety, and non-tumorigenicity. In addition, our study confirmed that USCs-Exos can alleviate kidney injury caused by DN, indicating that the protective effect of USCs is mainly based on its associated paracrine effect. Numerous studies have shown that circRNAs are stably and highly expressed in exosomes and participate in the progression of DN [25–27]. A recent study showed that extracellular vesicles derived from human umbilical cord MSCs improve the renal function of DN rats by promoting macrophage M2 polarization and reducing systemic and local renal inflammation [28]. The results of our study showed that USC-Exos had a protective effect on DN rats, and the protective effect of USC-Exos on DN rats was weakened after knocking down the circRNA ATG7 in exosomes. Moreover, our study demonstrated that the circRNA ATG7 can target miR-4500 and that miR-4500 can target SOCS1. Studies have shown that overexpression of SOCS1 can promote the repair of kidney injury in DN rats [29]. The STAT/SOCS1 signaling pathway is involved in macrophage M1 polarization and regulates various inflammatory events [30, 31]. Our research results indicate that the circRNA ATG7 regulates the SOCS1/STAT3 signaling pathway through miR-4500, thereby inhibiting the release of inflammatory factors and thus promoting macrophage M2 polarization.

Macrophages play an important role in podocyte injury in DN. For instance, HG-stimulated macrophage supernatant can generate podocyte damage, decreasing the expression of podocyte markers, reducing migration capacity and elevating apoptosis levels [32, 33]. Exosomes are membrane vesicles excreted by cells and liberated into the extracellular matrix. Macrophages can regulate inflammation by exosomes to impact wound healing and podocyte damage [34]. Our study certifies that the switching of macrophages from M1 to M2 alleviates DN progression.

In summary, USC-derived exosomal circRNA ATG7 can alleviate renal damage in DN rats and facilitate macrophage switching from M1 to M2 by regulating the SOCS1/STAT3 signaling pathway through miR-4500. Our research may apply novel therapy methods for DN that currently lack effective treatment, and circRNA ATG7 can serve as a latent remedial target for DN.

## Data Availability

The datasets used and/or analyzed during the current study are available from the corresponding author upon reasonable request.
